# Physician’s Complete Account-Book

**Published:** 1888-02

**Authors:** 


### PHYSICIANS' COMPLETE ACCOUNT BOOK. Published by G. W. Eschenbach. Easton, Pa.

This is one of the best small account books for visiting or office charges we have seen. It is divided into two parts—a visiting list proper, and a regular account book for making up bills This latter is indexed, increasing its convenience. Then there is the department of general memoranda The visiting list is especially useful for homœopathic physicians, as it contains spaces for writing the remedy given any patient at any date. Price, $1.00 and $2.25, according as bound.

The same publisher also issues the visiting list separately in a neat little book for the pocket, appropriately called "The Gem." Price, 35 cents.

